# Nitrosative Stress in the Frontal Cortex From Dogs With Canine Cognitive Dysfunction

**DOI:** 10.3389/fvets.2020.573155

**Published:** 2020-11-19

**Authors:** Sonja Prpar Mihevc, Maja Zakošek Pipan, Malan Štrbenc, Boris Rogelj, Gregor Majdič

**Affiliations:** ^1^Veterinary Faculty, Institute of Preclinical Sciences, University of Ljubljana, Ljubljana, Slovenia; ^2^Veterinary Faculty, Clinic for Reproduction and Large Animals, University of Ljubljana, Ljubljana, Slovenia; ^3^Department of Biotechnology, Jožef Stefan Institute, Ljubljana, Slovenia; ^4^Biomedical Research Institute (BRIS), Ljubljana, Slovenia; ^5^Department of Chemistry and Biochemistry, Faculty of Chemistry and Chemical Technology, University of Ljubljana, Ljubljana, Slovenia

**Keywords:** canine cognitive dysfunction, nitrosative stress, nNOS, eNOS, iNOS, 3-nitrotyrosine

## Abstract

Canine cognitive dysfunction (CCD) is an age-related disorder similar to human Alzheimer's disease (AD) that occurs in elderly dogs. Nitrosative stress has been implicated as one of the causes leading to neurodegenerative diseases, particularly AD. Its involvement in the development of CCD has not been studied so far. In the present study, immunohistochemical staining detected all three isoforms of nitric oxide synthases (nNOS, eNOS, and iNOS) and 3-nitrotyrosine (3-NT) in brains from CCD-affected dogs and non-demented control dogs in all layers of the canine frontal cortex. In CCD-affected and non-demented brains, nNOS was highly expressed in pyramidal-like neurons in the upper cortical layers. nNOS has also been observed in astrocytes in the CCD frontal cortex. The nNOS immunohistochemical staining was statistically significantly elevated in dogs with CCD in comparison to non-demented dogs. Blood vessel wall cells were positive for eNOS, which was also expressed in astrocytes and neurons. Intense 3-NT immunoreactivity was observed in the upper cortical layers, where amyloid-beta deposits spread in the last stage of CCD. Brain cells in the same area were highly immunoreactive for iNOS. This infers that neuroinflammation and nitrosative stress might exacerbate the neurodegenerative process in CCD-affected brains, ultimately leading to cognitive impairment.

## Introduction

Canine cognitive dysfunction (CCD) is a disease in many aspects similar to human Alzheimer's disease (AD) (1). CCD is common in aged dogs, and by 13 years of age, up to 60% of all dogs develop this illness (2). Dogs show cognitive deficits such as disorientation, memory loss, behavioral changes, and confusion (3). As in the pathophysiology of AD, the pathological hallmarks leading to the CCD are multifaceted, including the deposition of toxic amyloid-beta (Aβ), brain vascular damage, oxidative brain injury, mitochondrial dysfunction, excitotoxic neuronal damage, neuroinflammation, and cell death (1). Interestingly, neurofibrillary tangles (NFTs), aggregates of abnormally folded hyperphosphorylated protein TAU, which are almost always present in AD and are used for its diagnosis, were rarely observed in dogs (4–8). Nitrosative stress has been implicated as one of the causes leading to the development of neurodegenerative diseases, particularly Alzheimer's (9). However, its involvement in CCD has not been studied so far.

Nitric oxide (NO), produced by nitric oxide synthases (NOS), can be either neuroprotective or neurotoxic depending on its concentration and the redox state of the tissue (10, 11). It diffuses freely across membranes and regulates physiological processes in the central nervous system, where it acts as a vasodilator, inflammatory mediator, and neuromodulator (12), influencing cerebral blood flow, long-term potentiation, sleep–wake cycles, normal olfaction, and memory. There are three distinct NO synthases, namely, neuronal (nNOS, NOS1, type I), inducible (iNOS, NOS2, type II), and endothelial (eNOS, NOS3, type III). Both nNOS and eNOS are constitutively expressed and require increased intracellular Ca^2+^ for activation, while iNOS expression is induced in inflammatory cells (13, 14). Under physiological conditions, nNOS is expressed in the central and peripheral neurons and regulates neurotransmitters' release (15). eNOS is mostly present in endothelial cells (16), while iNOS can be expressed in many cell types in response to lipopolysaccharides, cytokines, and other agents promoting the neuroinflammation (9).

The expression of NOS isoforms was previously studied in the human AD brain (17–19). Upregulation of the iNOS has been detected in microglia and astrocytes during Aβ elicited inflammatory and immune responses (15, 20, 21). Under pathological conditions, NO can react with superoxide and form peroxynitrite. Peroxynitrite nitrates tyrosine residues in proteins to form 3-nitrotyrosine (3-NT) (9). Tyrosine nitration can induce structural changes leading to protein aggregation, which might also increase the propensity of Aβ to form amyloid plaques in AD (22). Nitroxidative damage can alter neurons and inflammatory and endothelial cells, thus causing changes in neuronal and inflammatory signaling and exacerbating the breakdown of the blood–brain barrier (23), all crucial in AD development and progression. Specifically, NO has been implicated in eliciting cellular changes and leading to somatic mutations. It can also affect cell cycle regulatory proteins, apoptosis, and DNA repair (16).

Murine models of AD that are deficient in iNOS due to genetic ablation are protected from premature mortality, cerebral plaque formation, increased Aβ levels, protein tyrosine nitration, astrocytosis, and microgliosis (15). iNOS inhibitor reduced Aβ-induced neurotoxicity by decreasing Aβ deposition and cognitive dysfunction in transgenic AD mice (22), indicating that nitrosative stress may be one of the key factors mediating Aβ pathogenesis in AD.

In the current study, we examined the protein expression of nNOS, eNOS, iNOS, and 3-NT, together with the presence of Aβ plaques, in CCD-affected and non-demented control canine brains using immunohistochemistry. In the frontal cortex, we investigated which cell types expressed the three NOS isoforms and 3-NT and whether the expression of nNOS, eNOS, iNOS, and 3-NT is changed in CCD brains.

## Materials and Methods

### Sample Preparation

The canine brains were obtained from privately owned dogs by owner consent after dogs were euthanized due to advanced dementia or other terminal conditions. The brains were collected by a veterinarian at the Institute of Pathology, Wild Animals, Fish and Bees (University of Ljubljana, Veterinary faculty) following the dog's owner's approval. One dog was already included in our behavioral study and diagnosed as having CCD based on the CADES (canine dementia scale) score (24). Others were presented only on the day of euthanasia and were considered affected by CCD if they displayed changes in behavior in at least three out of four domains: spatial orientation, social interactions, sleep–wake cycles, and house soiling. Furthermore, these dogs had no known record of other neurological or general illness in the past 6 months that could have caused similar symptoms. Before euthanasia, dogs were subjected to extensive physiological and neurological examination, and their blood and urine were analyzed to exclude metabolic diseases that could cause similar clinical signs. The CCD-affected dogs' average age was 16.20 ± 0.84 years (mean ± SD, *n* = 5). Brains of senior dogs without CCD symptoms or known behavioral changes in the last 3 years of their life were collected as control samples (non-demented aged controls; average age 13.25 ± 0.96 years, *n* = 4). The canine brains were dissected from the skull, and the left half of the brain was fixed in 4% paraformaldehyde at 4°C for at least 7 days to up to 1 month. The reason for this discrepancy in the fixation times is that the fixation regimens used on the first brains obtained were relatively short and, after consulting with pathologist, have been prolonged for optimal fixation. Afterward, pieces of the brain were embedded in the paraffin using an automated tissue processor (Tissue processor Leica TP 1020). Tissue blocks containing the frontal cortex area were cut to 7-μm-thick sections and further processed for immunohistochemistry. The frontal cortex was chosen as the area of interest since this is the region where Aβ deposits were most often observed in dogs aged 14–16 years in a previous study (6). The details on dogs that were used in this study are listed in Table 1.

**Table 1:** Information on dogs.

**Dog**	**Age (years)**	**Breed**	**Gender**	**Reason for euthanasia**
CCD1	16	Dachshund	M	Advanced CCD
CCD2	15	Pitbull terrier	F	Advanced CCD
CCD3	17	Mixed breed (medium)	F	Advanced CCD
CCD4	17	Mixed breed (medium)	M	Advanced CCD
CCD5	16	Shih-Tzu	F	Advanced CCD
OC1	14	German shepherd	F	Unable to stand up
OC2	14	Mixed breed (big)	M	Unable to stand up
OC3	12	Malteser	M	Heart failure
OC4	13	German shepherd	M	Unable to stand up

*CCD, canine cognitive dysfunction; OC, non-demented age-matched control*.

### Immunohistochemistry

After dewaxing, sections were subjected to antigen retrieval. For Aβ detection, they were first incubated in 98–100% formic acid for 20 s, followed by heat-induced antigen retrieval in sodium citrate buffer (10 mM sodium citrate and 0.05% Tween 20, pH 6) by boiling the slides for 10 min in a microwave oven. For all other proteins, only citrate buffer epitope unmasking was used. The staining was then performed with the Novocastra Novolink Polymer Detection System (Leica) according to the manufacturer's instructions. Primary antibodies used were against Aβ, nNOS, eNOS, iNOS, and 3-NT (Table 2). Brain sections were incubated with primary antibodies overnight at 4°C. The following day, secondary antibodies were added (Post Primary and Novolink Polymer), followed by incubation with the DAB chromogen. Sections were counterstained by hematoxylin and mounted with Pertex. Stained samples were visualized by bright-field microscopy (Nikon Eclipse 80i) and NIS elements software.

**Table 2:** Summary of primary antibodies.

**Target**	**Species raised/clonality**	**Dilution**	**Catalog number, source**
Aβ	Mouse monoclonal	1:1000	800712, BioLegend
nNOS	Rabbit polyclonal	1:1000	24287, ImmunoStar
eNOS	Rabbit polyclonal	1:500	ab3520, Abcam
iNOS	Rabbit polyclonal	1:100	06-573, Upstate
iNOS	Rabbit polyclonal	1:50	NB300-605, Novus Biologicals
3-NT	Rabbit polyclonal	1:200	06-284, Millipore
3-NT	Mouse monoclonal	1:200	MAB3248, R&D Systems
TUBB3	Mouse monoclonal	1:50	sc-80005, Santa Cruz Biotechnology
NEUN	Mouse monoclonal	1:200	MAB377, Millipore
GFAP	Mouse monoclonal	1:10000	G3893, Sigma-Aldrich
GFAP	Rabbit polyclonal	1:1000	HPA056030, Sigma-Aldrich
SMA	Mouse monoclonal	1:500	A5228, Sigma-Aldrich
NFH	Rabbit polyclonal	1:200	AB1989, Millipore

### Immunofluorescence

After dewaxing, sections were subjected to antigen retrieval as described in the previous section, followed by blocking of unspecific epitopes in 1.5% BSA, 10% normal goat serum, and 0.1% Triton X-100 in PBS for 60 min at room temperature. Sections were then incubated overnight at 4°C in the dark with different combinations of primary antibodies prepared in blocking solution. The primary antibodies used were the same as mentioned in the previous section. Additionally, antibodies against TUBB3, NEUN, GFAP, SMA, and NFH were used (Table 2). Next, the slides were washed and incubated with secondary antibodies [anti-rabbit Alexa Flour 488 and anti-mouse Alexa Fluor 594 or anti-mouse Alexa Fluor 488 and anti-rabbit Alexa Fluor 555 diluted 1:1000 in blocking buffer (all from Invitrogen)]. For negative controls, the primary antibodies were omitted, and only secondary antibodies were used. The nuclei were counterstained with DAPI (Sigma), followed by quenching of lipofuscin's autofluorescence by incubating sections in a 0.1% Sudan Black B solution prepared in 70% ethanol for 20 min. After washing, slides were mounted with ProLong Gold Antifade Mountant (Molecular Probes). Images were acquired with Zeiss LSM 710 inverted confocal laser scanning microscope with a Plan-Apochromat 63 × /1.4NA M27 oil immersion objective using immersion oil (Carl Zeiss). ZEN 2010 B SP1 software was used for image export and addition of scale bars.

### Quantification of Immunohistochemical Staining

Areas of the frontal cortex stained for nNOS, eNOS, iNOS, or 3-NT were captured on the Nikon Eclipse 80i microscope with a Nikon DS-Fi1 camera for quantitative analysis. The mean optical density for iNOS, eNOS, nNOS, and 3-NT was determined using the ImageJ software. First, DAB-specific images were generated from initial images by color deconvolution of each image by a color deconvolution plugin (25). Finally, the mean optical density was determined by ImageJ (26). Between 10 and 20 images were examined for each brain. They were captured randomly at 100 × magnification, and the average mean optical density and standard deviation were calculated for each group. Data were checked by the Kolmogorov–Smirnov test for normality, and statistical analysis was performed by one-way ANOVA followed by Bonferroni *post hoc* test. The difference was considered significant at *P* < 0.05.

## Results

### Clinical Status of Dogs

All dogs included in the study were presented to the veterinary clinics for various health problems (Table 1). Dogs showing changes in the behavior in at least three out of four domains—spatial orientation, social interactions, sleep–wake cycles, and house soiling—were considered affected by CCD. Older dogs without obvious signs of CCD were included in the control group. Mild elevations in renal and liver values were considered age-related, but the general hematologic parameters were normal.

### Aβ Pathology

**Figure 1:**
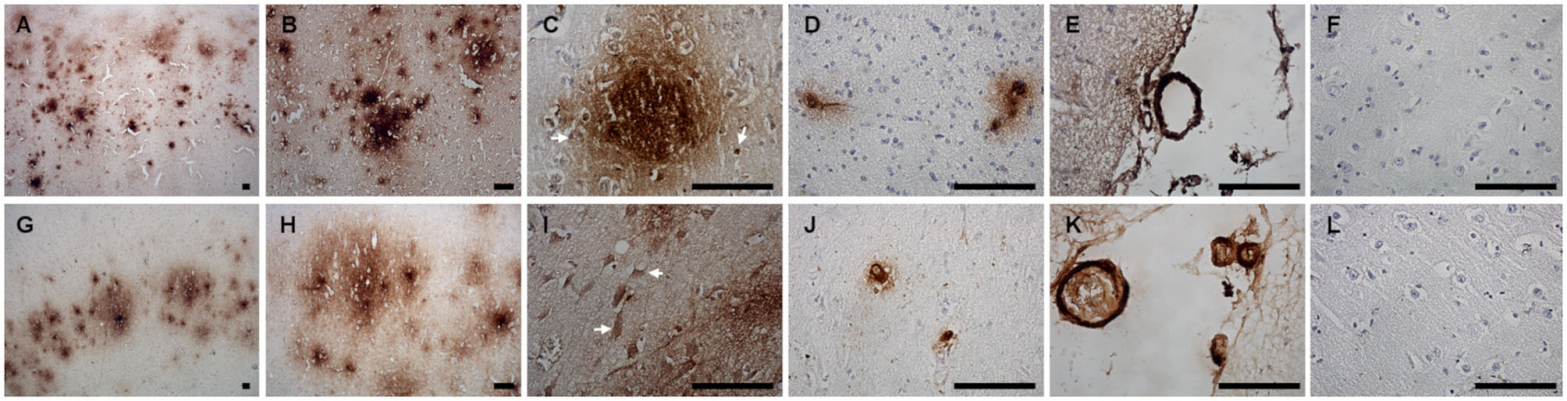
Amyloid β deposits in the canine frontal cortex. The frontal cortices of a 16-year-old dachshund with cognitive dysfunction **(A–F)** and a 14-year-old mixed breed non-demented dog **(G–L)** are shown. Dog with CCD exhibited predominately dense plaques distributed throughout all cortical layers **(A–C)**. The Aβ deposits in the frontal cortex of an old non-demented dog resided in deep cortical layers, occupied narrower cortical areas, and were diffuse and dense **(G,H)**. Intracellular Aβ was detected in deep cortical layers of CCD **(C)** and control brains **(I)**. Arrows point to the cells, probably neurons, harboring Aβ in their cytoplasm. Dense plaques with a concentrated core were observed in CCD brains **(C)**. All dogs examined had deposits of Aβ around some cortical blood vessels **(D,J)** and leptomeningeal blood vessels **(E,K)**. Hematoxylin-stained control images **(F,L)**. Scale bars are 100 μm.

Immunohistochemical staining against Aβ revealed that Aβ plaques are present in the brains of non-demented aged dogs and dogs with CCD. However, in most cases (4/5), Aβ plaques in superficial and deep cortical layers were more concentrated and denser and had a more delimited appearance in the brains of dogs with CCD (Figures 1A,B, Table 3) in comparison to non-demented old dogs (Figures 1G–I, Table 3). The Aβ deposits in the frontal cortex of the non-demented controls were observed mostly in deep cortical layers and were of the diffuse and dense types (Figures 1G–I). Several dense Aβ plaques contained a concentrated core (Figure 1C), which is often observed also in AD brains. Besides extracellular deposits, intracellular Aβ was detected in neuronal cytoplasm in deep cortical layers of CCD (Figure 1C) and non-demented brains (Figure 1I). All dogs with CCD and older dogs without the disease also had cerebral amyloid angiopathy (CAA; Table 3), which was evident as deposits of Aβ around the cerebral (Figures 1D,J) and leptomeningeal vessel walls (Figures 1E,K). Negative control images showed only hematoxylin stained nuclei (Figures 1F,L).

**Table 3:** Assesment of Aβ load.

**Dog**	**Cortical Aβ deposits**	**Cerebral amyloid angiopathy (CAA)**
CCD1	+++ (de)	++
CCD2	+++ (de)	+++
CCD3	+++ (de)	++
CCD4	+++ (de)	++
CCD5	+++ (di)	++
OC1	+++ (de)	+
OC2	++ (di, de)	+
OC3	++ (di, de)	++
OC4	++ (di, de)	++

*CCD, canine cognitive dysfunction; OC, non-demented age-matched control; CAA, cerebral amyloid angiopathy; +++, large cortical area occupied by Aβ plaques; de, dense and difuse Aβ plaques; di, mainly diffuse Aβ plaques; for CAA, the presence of Aβ-positive vessels was assessed, not the severity/grade of Aβ deposition*.

### nNOS

**Figure 2:**
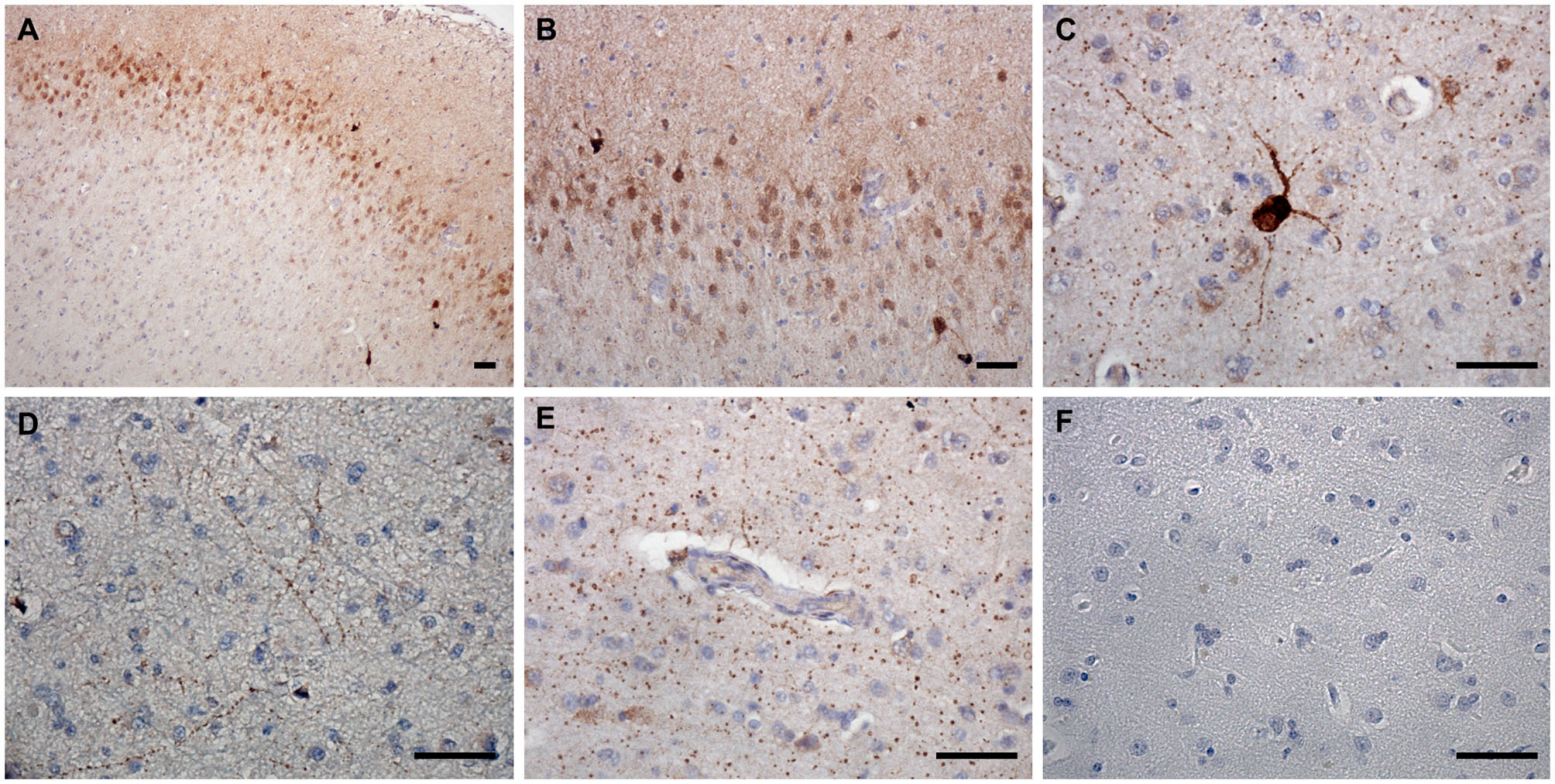
nNOS expression in the canine frontal cortex. The frontal cortex of a 16-year-old Shih-Tzu with canine cognitive dysfunction is shown. Immunoreactivity of nNOS was most prominent in the cytoplasm and the nuclei of cells in the upper cortical layers **(A,B)**. Some neurons in deep cortical layers were highly positive for nNOS, which was distributed in their somas and processes **(A,C)**. In the deep cortical layers, the neuronal axons positive for nNOS were frequently observed **(D)**. nNOS was also detected in axons around blood vessels with similar immunoreactivity pattern as in the surrounding parenchyma **(E)**. Hematoxylin-stained control image **(F)**. Scale bars are 50 μm.

nNOS expression was detected in superficial and deep cortical layers and in the axons in the white matter in frontal cortices of CCD-affected and non-demented control brains (Figure 2). The neuronal somas and the neuronal process were immunoreactive in the gray matter (Figures 2A–D). nNOS was detected in the cytoplasm and also showed plasmalemmal and nuclear localization (Figures 2A–C). Individual neurons in deep cortical layers were highly positive for nNOS (Figure 2C) and axons positive throughout the parenchyma (Figure 2D). nNOS was observed in axons surrounding blood vessels with a similar staining pattern in the surrounding parenchyma (Figure 2E).

**Figure 3:**
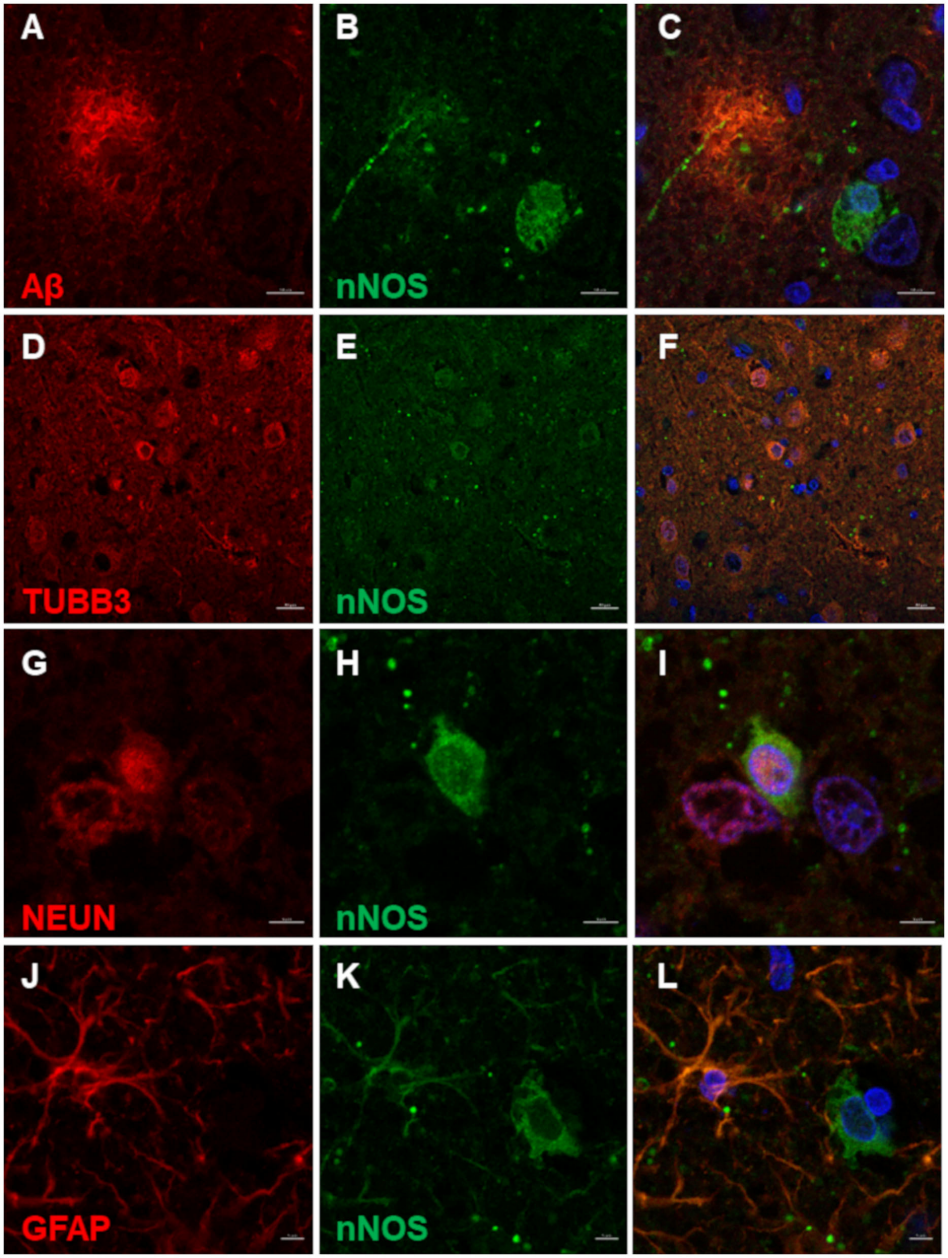
nNOS immunofluorescence in the canine frontal cortex. The frontal cortex of a 16-year-old dachshund with canine cognitive dysfunction is shown. nNOS cytoplasmic staining was detected in neuronal somas and processes around Aβ plaques **(A–C)**. Neurons labeled by TUBB3 **(D)** and NEUN **(G)** were immunoreactive for nNOS in the superficial cortical layers **(D–F)** and deep cortical layers **(G–I)**. Some astrocytes, marked by GFAP **(J)**, were immunopositive for nNOS **(K,L)**. The third column shows merged images of the first two images of each line. Nuclei were counterstained with DAPI. Scale bars are 10 μm in **(A–C)**, 20 μm in **(D–F)**, and 5 μm in all other images.

nNOS reactive cells surrounded Aβ deposits (Figures 3A–C). Co-immunolabeling confirmed the neuronal expression of nNOS as the TUBB3 and NEUN marked neurons also expressed nNOS (Figures 3D–I). Interestingly, some nNOS-expressing astrocytes were detected by the co-localization of nNOS and astrocytic marker GFAP (Figures 3J–L).

### eNOS

**Figure 4:**
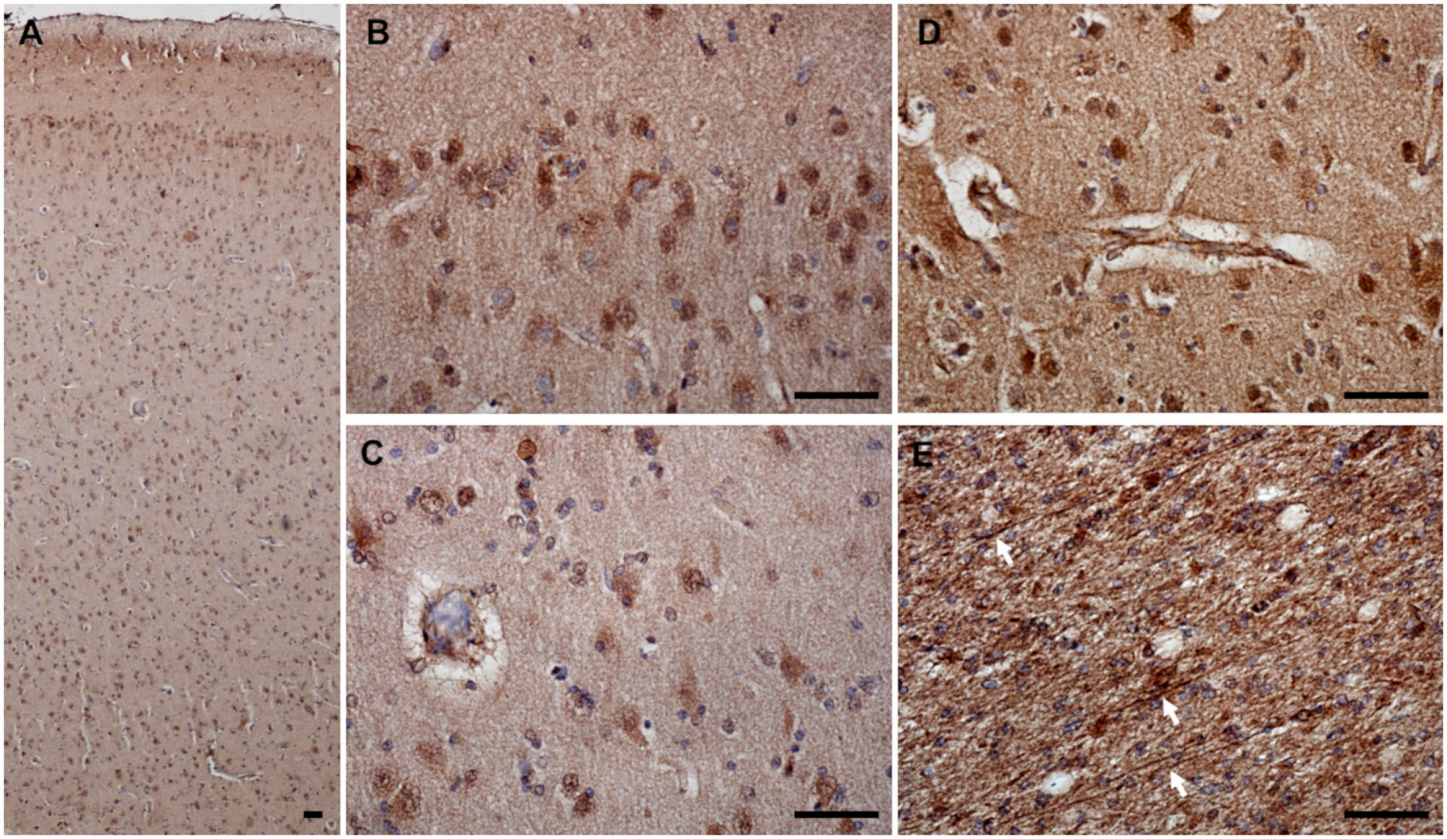
eNOS expression in the canine frontal cortex. The frontal cortex of a 16-year-old Shih-Tzu with canine cognitive dysfunction is shown. eNOS-expressing cells were detected in all cortical layers **(A)**. eNOS was noticed in the cytoplasm and nuclei of cells in superficial **(B)** and deep **(C)** cortical layers. Blood vessels were positive for eNOS in all cortical layers. Image showing the blood vessel in the deep cortical layer **(D)**. eNOS was also detected in the axons in the white matter [arrows pointing to individual eNOS-positive axons, **(E)**]. Scale bars are 50 μm.

eNOS was expressed in all brain samples and was found in the superficial and deep cortical layers (Figures 4A–D) as well as in the axons in underlying white matter (Figure 4E). Based on cellular morphology, eNOS was expressed in astrocytes, neurons, neuronal process, microglia, and around blood vessels (endothelial) both in CCD and non-demented control brains. Since the staining pattern was similar for all brains, only representative photomicrographs of one brain are shown (Figure 4). Besides cytoplasmic expression, the nuclear expression of eNOS was also detected (Figures 4B–D). Endothelial expression of eNOS was evident in the cytoplasm of blood vessel wall cells (Figure 4D).

**Figure 5:**
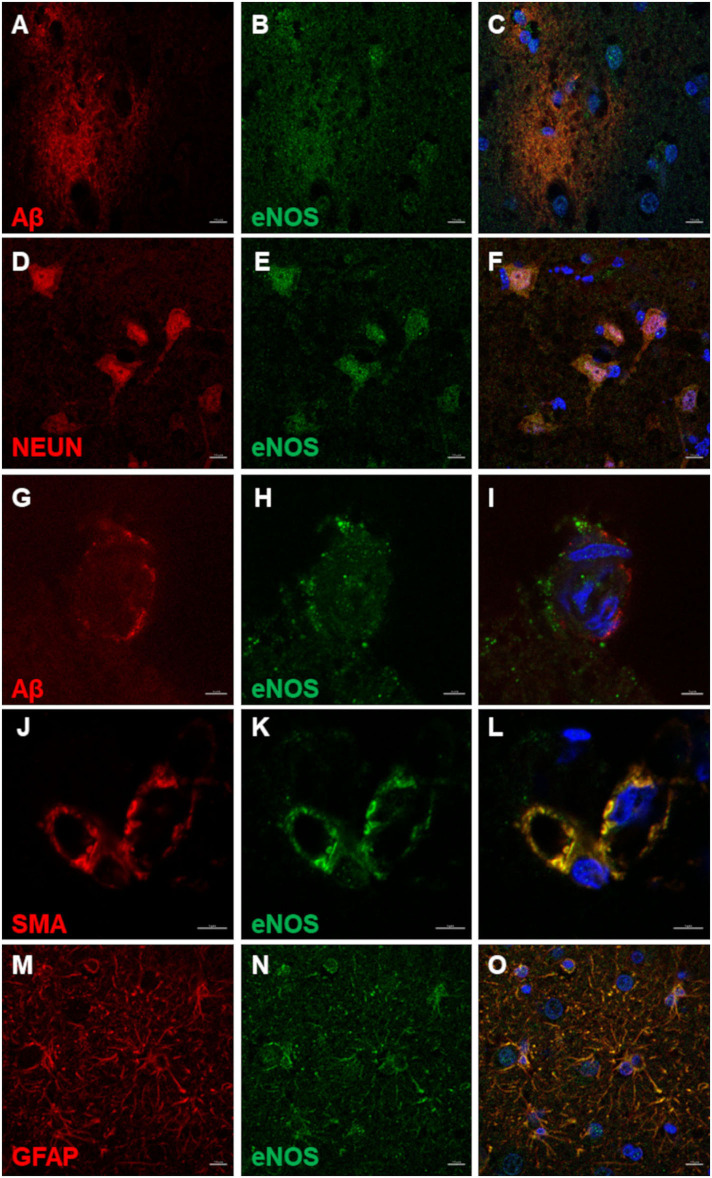
eNOS immunofluorescence in the canine frontal cortex. The frontal cortex of a 16-year-old dachshund with canine cognitive dysfunction is shown. eNOS immunoreactive cells were detected in and around Aβ plaques **(A–C)**. Neurons positive for NEUN **(D,F)** harbored eNOS in their nuclei and cytoplasm **(E,F)**. eNOS-positive cells in blood vessel walls **(H,I)** were noticed in some vessels surrounded by Aβ deposits **(G,I)**. The vascular smooth muscle cells, immunoreactive for SMA (smooth muscle actin) **(J,L)**, expressed eNOS **(K,L)**. eNOS was highly expressed in astrocytes **(N,O)**, marked by GFAP **(M,O)**. Images **(C,F,I,L,O)**, are merged images of two preceding images in the same line. Nuclei were counterstained with DAPI. Scale bars are 10 μm in **(A–F)** and **(M–O)** and 5 μm in all other images.

Nuclear and perinuclear expression of eNOS in cells in the amyloid plaque area and surrounding the plaques was observed (Figures 5A–C). In neurons, marked by NEUN, eNOS was expressed in the nuclei and in the cytoplasm (Figures 5D–F). In the CAA-affected blood vessels, eNOS immunoreactivity did not co-localize with Aβ staining (Figures 5G–I). Aβ deposits seemed to load at the basement membrane and were surrounding eNOS-positive blood vessel wall cells. These cells were SMA positive, presumably vascular smooth muscle cells (Figures 5J–L). eNOS was also expressed in astrocytes, detected with co-localization of eNOS and GFAP (Figures 5M–O).

### iNOS

**Figure 6:**
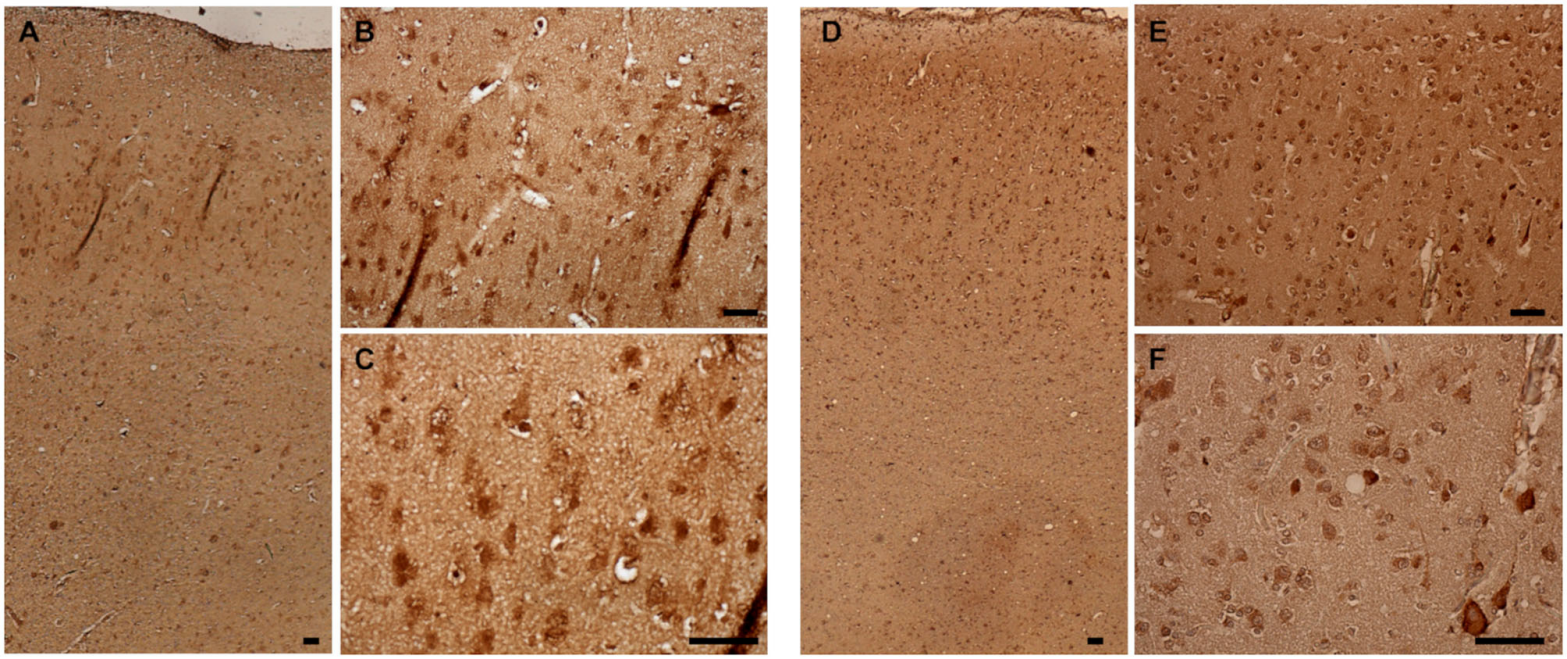
iNOS expression in the canine frontal cortex. The frontal cortices of a 16-year-old dachshund with cognitive dysfunction CCD **(A–C)** and a 14-year-old mixed breed non-demented dog **(D–F)** are shown. iNOS immunoreactivity was present in all cortical layers. Cytoplasmic and nuclear iNOS staining was prominent in the cells in the upper cortical layers of the CCD frontal cortex **(A–C)**. Also, in the aged non-demented dog, the iNOS reactivity was present, although it seemed more uniform throughout the cortical layers **(D)**. In upper cortical layers of the non-demented dog, some neuronal somas displayed intense iNOS staining, whereas others were faintly stained **(E,F)**. Scale bars are 50 μm.

**Figure 7:**
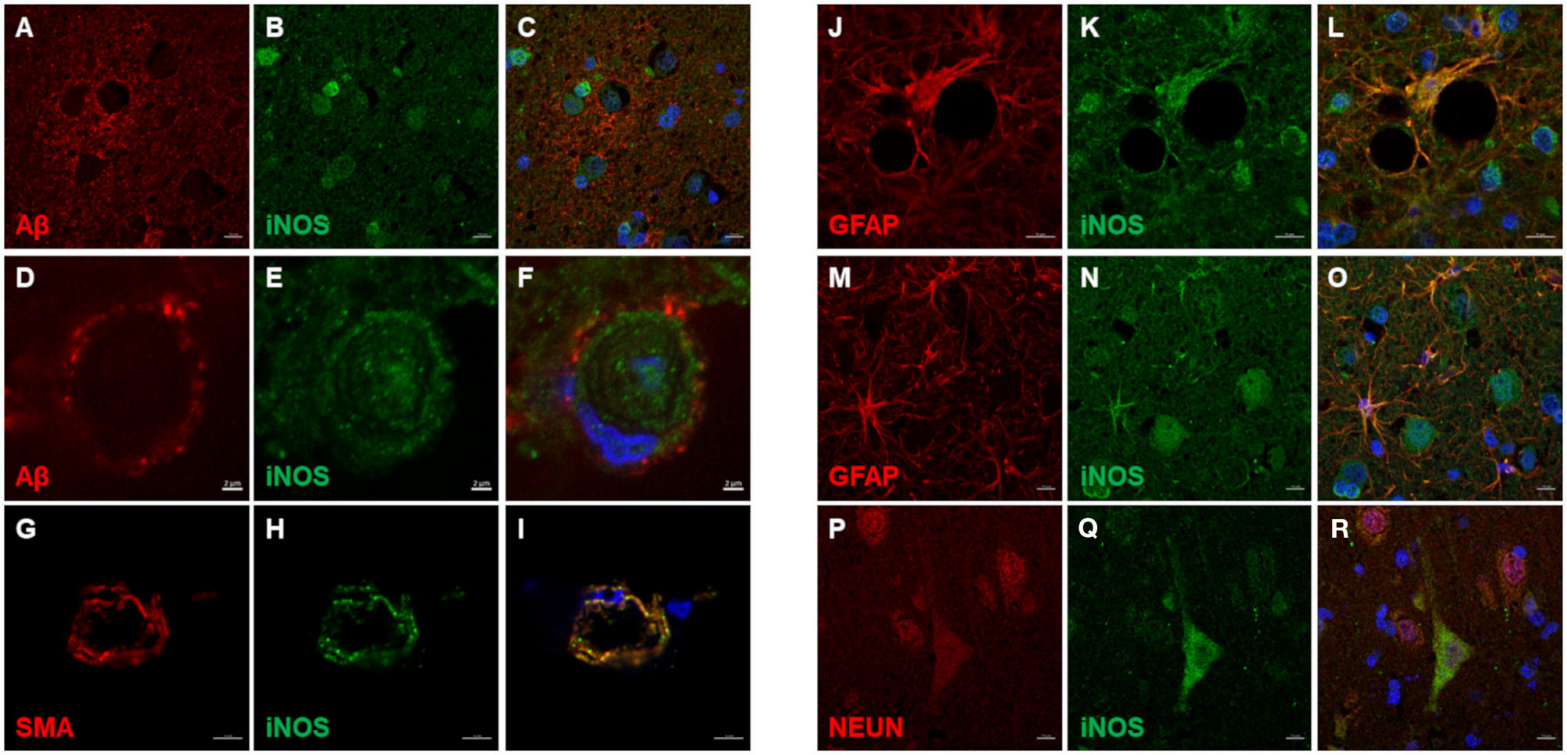
iNOS immunofluorescence in the canine frontal cortex. The frontal cortex of a 16-year-old dachshund with canine cognitive dysfunction is shown. iNOS highly immunoreactive cells were detected in and around Aβ plaques **(A–C)**. Some cerebral amyloid angiopathy (CAA) affected blood vessel **(D,F)** showed iNOS staining in the vessel walls **(E,F)**. Vascular iNOS-expressing cells **(H,I)** were smooth muscle actin (SMA) positive **(G,I)**. iNOS **(K,L,N,O)** was highly expressed in GFAP-positive astrocytes **(J,L,M,O)**, which were densely clustered around blood vessel walls **(J–L)**. iNOS was also detected in neuronal cytoplasm [iNOS - neuronal marker **(Q,R)**, NEUN **(P,R)**]. Images **(C F,I,L,O,Q)**, are merged images of two preceding images in the same line. Nuclei were counterstained with DAPI. Scale bars are 2 μm in **(D–F)**, 5 μm in **(G–I)**, and 10 μm in all other images.

iNOS immunoreactive cells were detected in all cortical layers of the CCD brain (Figures 6A–C) and the non-demented age-matched brain (Figures 6D–F). iNOS expression was detected in astrocytes, neuronal processes, pyramidal neurons, endothelial cells, and vascular smooth muscle cells (Figures 6, 7). Intense iNOS staining was noticed immediately below the pial surface (Figures 6A,D) and was more abundant in the upper cortical layers. This expression seemed to be more prominent in CCD-affected brains (Figures 6A–C). iNOS expression was detected in the cytoplasm, periplasm, and cell nuclei (Figures 7A–C). iNOS-positive cells in the blood vessel walls did not co-localize with Aβ deposits (Figures 7D–F). Vascular smooth muscle cells expressed iNOS in CAA-affected blood vessels (Figures 7G–I). Astrocytes with high expression of iNOS were surrounding blood vessels (Figures 7J–L). Double immunostaining revealed astrocytes that were positive for both GFAP and iNOS (Figures 7M–O) and neurons immunopositive for NEUN and iNOS (Figures 7P–R).

### 3-NT

**Figure 8:**
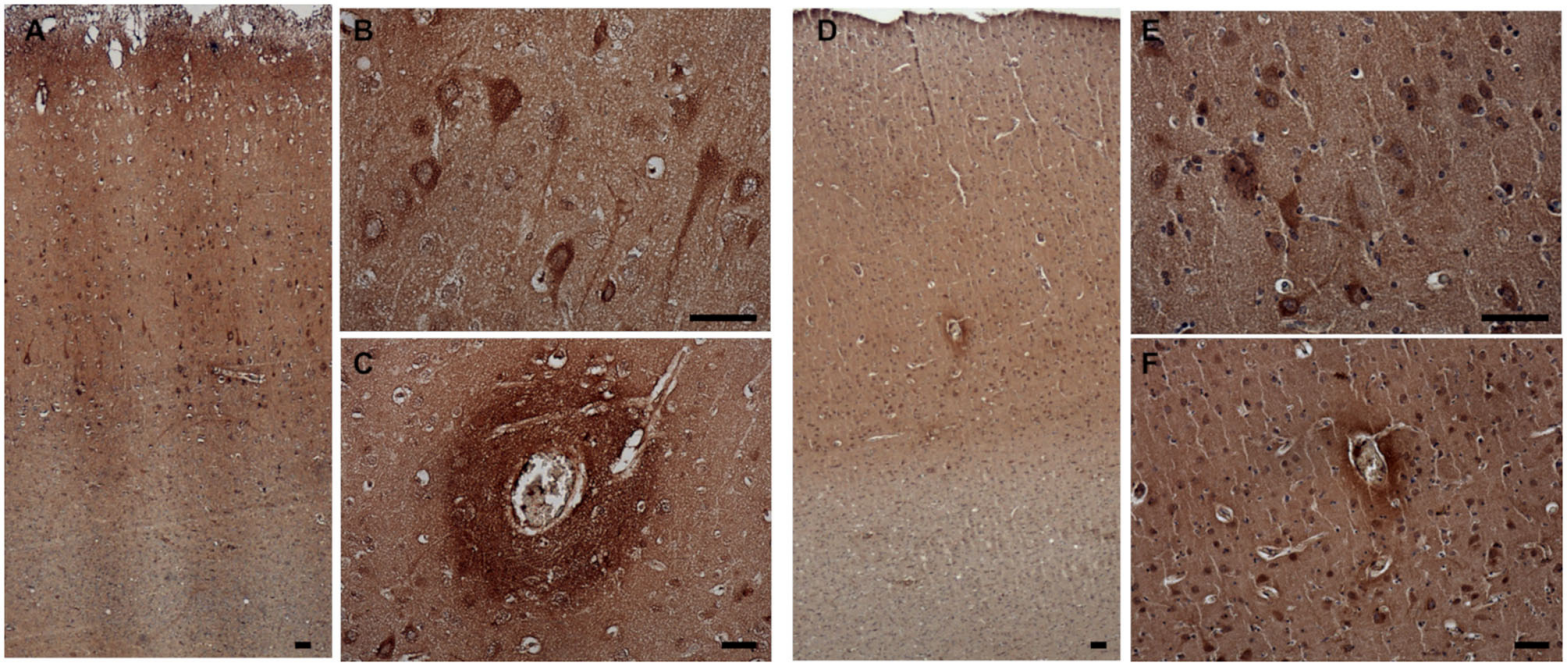
3-NT expression in the canine frontal cortex. The frontal cortices of a 16-year-old dachshund with cognitive dysfunction CCD **(A–C)** and a 13-year-old non-demented German shepherd **(D–F)** are shown. 3-NT immunoreactivity was detected in all cortical layers. In the CCD-affected brain, intensive 3-NT neuronal staining was present **(B)**. Some areas around blood vessels were strongly 3-NT immunopositive, forming perivascular “collars” in demented **(C)** and non-demented brains **(F)**. Also, in the aged non-demented frontal cortex, 3-NT was present in the cellular nuclei and cytoplasm **(E,F)**. Scale bars are 50 μm.

**Figure 9:**
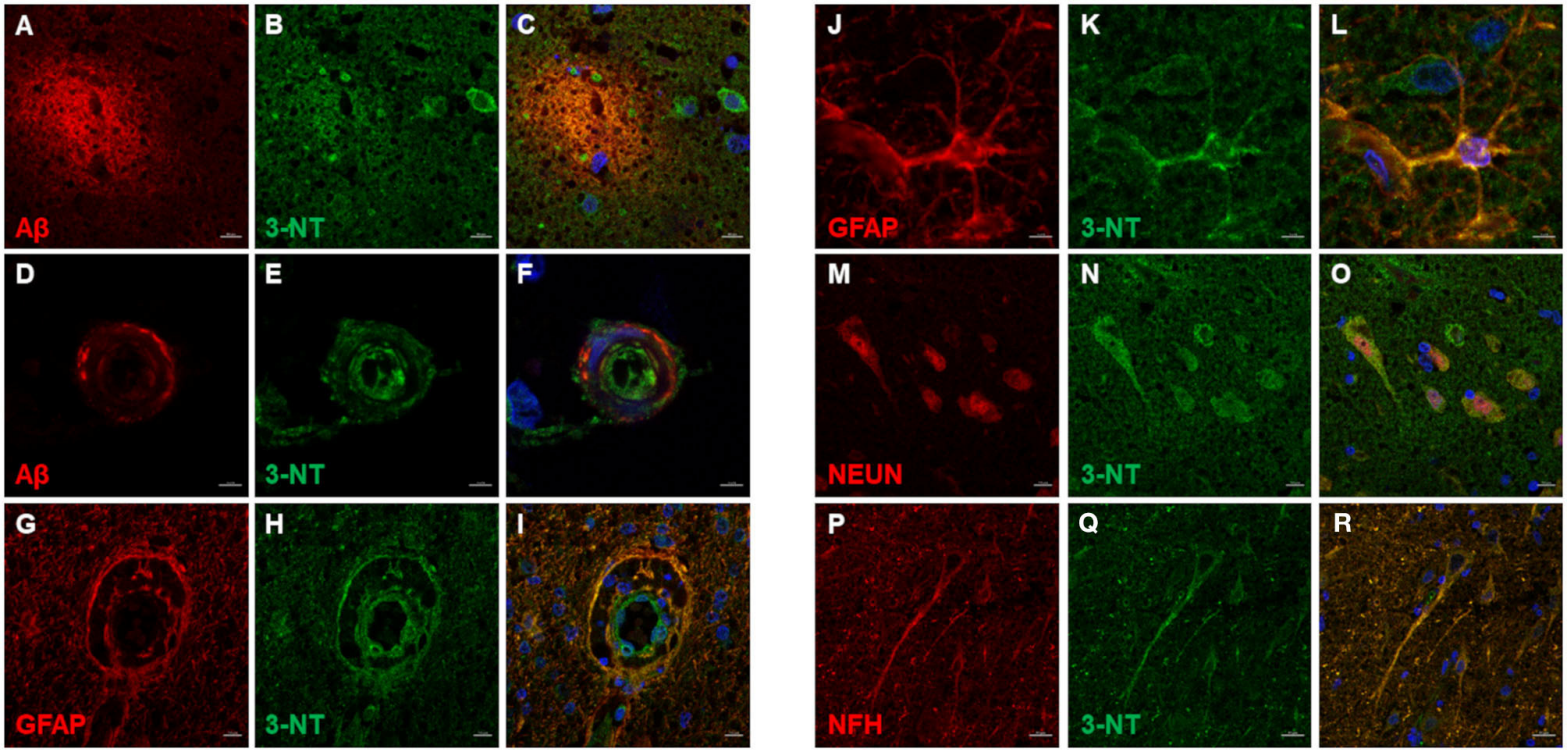
3-NT immunofluorescence in the canine frontal cortex. The frontal cortex of a 16-year-old dachshund with canine cognitive dysfunction is shown. 3-NT highly immunoreactive cells were detected in and around Aβ plaques **(A–C)**, and parenchymal 3-NT staining was observed **(B,C)**. Cerebral amyloid angiopathy (CAA)-affected blood vessel **(D,F)** showed 3-NT staining **(E,F,H,I)**. 3-NT was highly expressed in GFAP-positive astrocytes **(G–I,J–L)**, clustered around blood vessel walls **(G–I)**. 3-NT was detected in neuronal cytoplasm and nuclei **(N,O)** [NEUN marks neurons **(M,O)**] and neuronal process **(Q,R)** [NFH marks neurons **(P,R)**]. Images **(C F,I,L,O,R)**, are merged images of two preceding images in the same line. Nuclei were counterstained with DAPI. Scale bars are 5 μm in **(D–F)** and **(J–L)**, 20 μm in **(P–R)**, and 10 μm in **(A–C,G–I,M–O)**.

3-NT immunoreactivity was present both in the gray and the white matter of CCD (Figures 8A–C) and non-demented control brains (Figures 8D–F). In the demented canine frontal cortex, 3-NT immunostaining appeared more intense (Figures 8A–C) in comparison to non-demented controls (Figures 8D–F). Besides 3-NT-positive cells, the whole brain parenchyma was diffusely stained for 3-NT (Figure 8). Neurons in the upper cortical layers showed high cytoplasmic 3-NT immunoreactivity in the CCD brain (Figures 8A,B) and in some upper cortical regions of the non-demented brain (Figure 8E). Around some blood vessels, perivascular “collars” of 3-NT (intense 3-NT staining surrounding the vessel) were detected in demented (Figure 8C) and non-demented brains (Figure 8F). 3-NT was detected in the cytoplasm and the nuclei of cells surrounding Aβ deposits (Figures 9A–C). Plaque areas were diffusely stained for 3-NT (Figure 9B). 3-NT was present around and in the cells composing the CAA-affected blood vessels (Figures 9D–I), particularly in the endothelial cells facing the blood vessel lumen and astrocytes surrounding the blood vessel walls (Figures 9D–I). Parenchymal GFAP-positive astrocytes also contained 3-NT (Figures 9G–I,J–L), as well as neurons that were positive for NEUN (Figures 9M–O) and NFH (Figures 9P–R).

### Quantification of Immunohistochemical Staining

**Figure 10:**
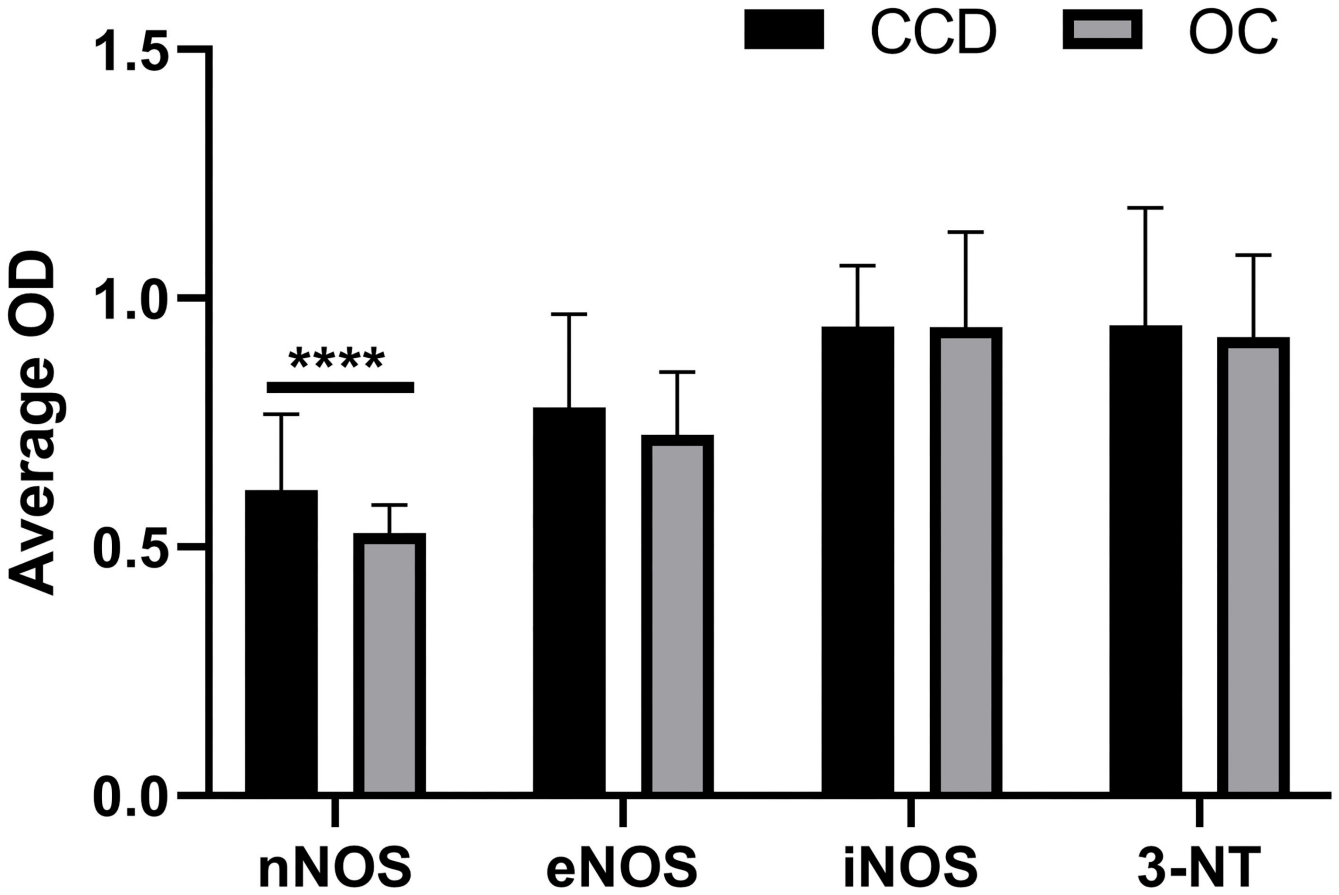
Quantification of immunohistochemical staining of NOS isoforms and 3-NT. The mean optical density (OD) was measured on randomly captured images of the frontal cortex of dogs with CCD (CCD; *n* = 5) and non-demented aged control dogs (OC; *n* = 4). Average OD measurements and standard deviations are shown for nNOS, eNOS, iNOS, and 3-NT. For the individual frontal cortex, 10–20 images were quantified. The results are statistically significant for nNOS (^****^P < 0.0001).

The immunoreactivity of the NOS isoforms and the 3-NT in frontal cortices was quantified. The optical density (OD) measurements revealed a slightly elevated, although not statistically signifcant, average OD for eNOS, nNOS, and 3-NT in the frontal cortex from demented compared to non-demented dogs (Figure 10). The average OD for iNOS was almost equal for both groups. In the case of nNOS, the difference was statistically significant (*P* < 0.0001) (Figure 10). The average OD was higher for iNOS and 3-NT compared to OD for nNOS and eNOS.

## Discussion

Aging causes major alterations of all components of the neurovascular unit and compromises brain blood supply. It is also associated with a loss of antioxidative capacity and NO-dependent peroxynitrite formation, which nitrates the tyrosine residues on proteins, forming 3-nitrotyrosine. Some human studies suggest the neurotoxic role of NO and report elevations in the NOS isoforms expression during AD progression (21, 27), whereas others describe reductions in NOS expression with aging (18, 28).

Aged dogs naturally develop a broad spectrum of neuropathology and neurobiological changes that parallel observations in aging humans (1). In the current study, we investigated immunoreactivity of the three NOS isoforms (nNOS, eNOS, and iNOS) and 3-NT in brains of dogs with CCD and non-demented aged controls, along with the expression of pathological Aβ. There are almost no data on the expression of the three NOS isoforms and the presence of 3-NT in the canine brain. One study explored the expression of iNOS in dogs infected with canine distemper virus, with iNOS immunopositive staining observed in astrocytes in the cerebellar white matter (29).

We detected the nNOS, eNOS, and iNOS in the cytoplasm and plasmalemma and in the cells' nuclei in the canine frontal cortices. nNOS was expressed in all cortical layers, but most prominently in the somas and process of neurons with pyramidal-like morphology in the upper cortical layers. Previously, nNOS was found aberrantly expressed in pyramidal-like cortical neurons in human AD brains (30). We also observed strongly immunoreactive large multipolar-like neurons, which were present in upper and deep cortical layers. Such neurons expressing nNOS were also previously observed in human AD-affected brains (17). In the frontal cortex from dogs with CCD, nNOS-positive neurons were often located in the vicinity of Aβ plaques. In AD transgenic mice, the Aβ plaques were closely associated with dystrophic nNOS-positive neurons, while nNOS-expressing neurons located more distal to plaques appeared to be unaffected (31). The expression of nNOS was suggested to be a response to plaque-mediated damage to neurons (31). Thus, Aβ mediated induction of nNOS might have a role in the pathogenesis of CCD, as we also noticed statistically significantly elevated expression of nNOS in dogs with CCD in comparison to non-demented aged dogs. Moreover, cytoplasmic astrocytic nNOS expression was also observed in CCD-affected brains. Similarly to our finding, nNOS immunoreactivity in astrocytes was previously reported in human patients with AD (32).

Upregulation and/or *de novo* expression of the nNOS commonly occurs in diverse neurodegenerative diseases, including AD (33). The recruitment of NO might have a compensatory role to boost synaptic transmission and plasticity during the early stages of AD when a moderate loss of neurons and consequently of brain function is present (34). However, nNOS-mediated nitrosative stress was also connected to early cognitive/motor deficits due to synaptic loss and negatively regulated neurogenesis (35, 36).

Although the precise role of nNOS upregulation in neurodegenerative diseases is still under debate, several studies have reported an upregulation of nNOS in various parts of the brain, including hippocampal pyramidal neurons in AD (30, 32). Consistent with this, another study showed that the proteome of hippocampal neurons has an up-regulated expression of the adaptor protein essential for the proper function of nNOS (37). In the present study, the nNOS levels, deduced from OD measurements of immunohistochemically stained frontal cortex sections, were also elevated in the brains from dogs with clinical signs of CCD. This suggests a possible involvement of increased nNOS expression in CCD's pathogenesis, although further studies with more dogs will be needed to confirm this finding conclusively. We further looked at the distribution of nNOS-positive nerve fibers around blood vessels, which did not appear any different to the staining pattern of the fibers further away from the vessels. However, at a closer look, there might have been some immunoreactivity in the blood vessel wall. In-depth studies are needed to corroborate this assumption. Nevertheless, detection of nNOS immunoreactive fibers in the walls of canine arteries previously provided evidence that blood vessels are innervated by nitrergic nerves (38), and these perivascular nerves containing NOS are crucial in eliciting the neurally induced, NO-mediated arterial relaxation (39). There is scarce information on how age affects NO release and the vascular tone, and if nNOS and eNOS contribute to it, since all neurovascular unit components, i.e., endothelial and smooth muscle cells, as well as perivascular neurons are altered by aging.

Cytotoxic effects of NO in patients with AD are only observed once NO is converted to peroxynitrite (40). The presence of peroxynitrite-mediated damage in the AD brain is common and is caused by increased protein nitration inside the neurons (41). In our study, immunoreactivity for 3-NT in CCD-affected brains was intense, with 3-NT detected in neurons, astrocytes, and blood vessels. 3-NT was also detected extracellularly throughout the brain parenchyma, including the areas of Aβ plaques. The consequences of Aβ nitration are an enhanced tendency to aggregate and seed (22). The nitrated Aβ also damaged synaptic function and memory in APP/PS1 mice (22).

Furthermore, some Aβ aggregates were also shown to induce NO formation (42). The neurons in the upper cortical layers of the canine frontal cortex displayed intense 3-NT immunoreactivity, with the 3-NT present in their somas and process. Similarly, 3-NT was detected in senile plaques, pyramidal neurons, astrocytes, and blood vessels in the human AD brain (19). There was also “collar”-like staining present around several blood vessels in all cortical layers in CCD and non-demented aged brains, possibly inferring to disruption of the blood–brain barrier. 3-NT was also present in the brains from non-demented aged dogs, although qualitative observations did suggest a higher presence of 3-NT in some areas in brains from dogs with CCD. However, when quantified by measuring the optical density of the staining, there was no statistically significant difference between CCD and control dogs. This does not exclude the possibility of regional differences in the presence of 3-NT. However, we choose view fields randomly for optical density measurement, which could mask the small regional difference in the 3-NT immunodetection.

The distribution of Aβ within the frontal cortex followed a specific age-related pattern that was previously described (6, 7, 43). In concurrence with findings by Schmidt et al. (6), the CCD-affected dogs exhibited diffuse plaques in deep and dense core plaques in superficial cortical layers. In contrast, most of the non-demented aged dogs appeared to possess both types of plaques in a narrower area in deep cortical layers. The smaller compact dense Aβ deposits were the predominant type in the CCD frontal cortex. This distribution pattern of canine plaques resembled those seen in humans (44, 45). The close-up images of the dense Aβ plaques also indicated the presence of a core. Their composition would need to be studied with advanced methods, like in the AD (46). The intraneuronal Aβ was noticed in CCD and non-demented aged brains. These neurons burdened with increasing levels of soluble and oligomeric Aβ, which are known to be the most toxic amyloid species within the brain, were recently proposed to produce cytokines inflicting inflammation in the AD pathogenesis (47). Vessel-associated Aβ deposits characteristic of CAA were observed in the parenchyma and leptomeninges of the frontal cortices of CCD and non-demented aged dogs. This is in order with a previous study that found amyloid accumulation in the cerebral blood vessels' walls predominantly in the canine frontal cortex (48).

In CCD-affected brains, Aβ deposits were present around blood vessel walls at the basement membranes directly above endothelial cells. Some cortical and leptomeningeal blood vessels surrounded by Aβ were also immunopositive for 3-NT, iNOS, and eNOS. In the present study, eNOS was expressed in the blood vessels' endothelial cells and in neurons and astrocytes. Although we expected eNOS to be expressed only in the endothelial cells, a similar expression pattern was described before in human AD brains (49, 50), suggesting that eNOS expression is not limited to endothelial cells in diseased brains. In primary neurons, eNOS was localized in dendritic spines (51). In CCD and non-demented aged brains, eNOS immunoreactive cells were detected around Aβ plaques; some of these were neurons that expressed eNOS in their cytoplasm and nuclei. Astrocytes and vascular smooth muscle cells were also immunopositive for eNOS. We did not observe any significant differences between the mean OD for eNOS measured for the CCD and non-demented group. However, decreased eNOS is a common feature of aging and cerebrovascular disease. It has been suggested that in AD patients, eNOS deficiency could result in hypoperfusion (52). Consequently, the blood flow to the brain is reduced dramatically, and the clearance of Aβ protein could be negatively affected. One study in mice has associated chronic loss of endothelial NO as an important contributor to both Aβ-related pathology and cognitive decline (53). In CAA, eNOS deficiency first leads to increased cerebrovascular concentration of Aβ along with compensatory mechanisms to protect the vasculature (54). NO, produced by eNOS, is an important vasodilator (16), and this role has also been demonstrated in canine cerebral arteries *ex vivo* (55).

It is unclear whether high levels of NO, generated during inflammation, are due to eNOS or iNOS activity, but are usually attributed to the iNOS (9). NO, produced at high levels, acts as a pro-inflammatory molecule causing nitrosative and oxidative stress, whereas, under physiological conditions, low levels of NO have homeostatic properties (10). We observed intense iNOS staining in the upper cortical layers of the frontal cortex, mostly in neurons and astrocytes. Double immunofluorescence staining showed that cells surrounding Aβ deposits were intensely iNOS positive, but so were the astrocytes surrounding the blood vessels, the blood vessel wall cells, and the intraparenchymal neurons and astrocytes. Interestingly, the superficial cortical layers are the areas where the Aβ plaques spread to during the last stage of the disease. Thus, the cytoplasmic and nuclear iNOS-expressing cells surrounding Aβ deposits might be part of a pro-inflammatory response to Aβ. In AD, it was suggested that elevated levels of iNOS are responsible for promoting neurodegeneration by inducing oxidative and nitrosative damage (56). During Aβ-elicited inflammatory and immune responses, iNOS expression is increased in microglia and astrocytes (15, 17, 20, 57).

Although based on the visual observations of immunohistochemically stained sections of the frontal cortex, there seemed to be differences in the expression/presence of the three NOS isoforms and 3-NT, the quantifications did not confirm these except for nNOS. A clear limitation of the present study is the low number of cases. Furthermore, the quantification of the immunohistochemistry with DAB chromogen is not the optimal method, since the time of the exposure to the chromogen itself influences the intensity of the staining reaction. Although we tried to meticulously time the exposure of the tissue sections to the chromogen, just a few seconds might have made a difference in the intensity of the signal along with the regional differences in the expression due to different cellular compositions and hence immunogenicity. More sensitive methods, like proteomic analysis, might shed light on the involvement of nitrosative stress in CCD's pathogenesis in the future. Based on the similarity of the expression pattern of the three NOS isoforms and the nitrosative stress marker 3-NT between AD and CCD-affected brains, nitrosative stress might also play a role in CCD.

Nevertheless, this study provides the first investigation of the immunolabeling patterns of nNOS, eNOS, iNOS, and 3-NT in CCD-affected and non-demented canine brains, suggesting that nitrosative stress and inflammation might have a role in the pathogenesis of CCD. Whether the nitrosative, oxidative changes and neuroinflammation are causing CCD or are just age-related conditions that might exacerbate the Alzheimer's-like pathology in CCD is still uncertain. However, dogs suffering from CCD represent a spontaneous animal model of dementia, which more faithfully recapitulates the complexities of sporadic human AD than transgenic rodent models. Therefore, modulators of NO signaling, for instance, nitric oxide synthase inhibitors, could be tested for managing the CCD, and such an experiment could pave the way to develop valuable treatments for dogs and humans with dementia.

## Data Availability Statement

The raw data supporting the conclusions of this article will be made available by the authors, without undue reservation.

## Ethics Statement

Ethical review and approval was not required for the animal study because the canine brains were obtained from privately owned dogs by owner consent after dogs were euthanized due to advanced dementia or other terminal conditions. Therefore, no approval of the ethical committee was needed according to the Slovenian legislation and official opinion from the Administration of the Republic of Slovenia for Food Safety, Veterinary, and Plant protection, responsible for issuing ethical permits for animal experiments. Written informed consent for participation was not obtained from the owners because verbal consent for participation was obtained from the animal's owners.

## Author Contributions

SP: conceptualization, formal analysis, funding acquisition, investigation, project administration, resources, supervision, visualization, roles/writing—original draft, writing—review, and editing. MZ and MŠ: resources, writing—review, and editing. BR: funding acquisition, resources, writing—review, and editing. GM: conceptualization, formal analysis, funding acquisition, project administration, resources, supervision, roles/writing—original draft, writing—review, and editing. All authors read, edited, and approved the final manuscript.

## Conflict of Interest

The authors declare that the research was conducted in the absence of any commercial or financial relationships that could be construed as a potential conflict of interest.
